# Bedside and laboratory diagnostic testing in myasthenia

**DOI:** 10.1007/s00415-022-10986-3

**Published:** 2022-02-10

**Authors:** Katie Yoganathan, Alexander Stevenson, Awais Tahir, Ross Sadler, Aleksandar Radunovic, Naveed Malek

**Affiliations:** 1 University of Oxford, UK and Oxford University Hospitals, Oxford, UK; 2 Department of Neurology, National Hospital for Neurology and Neurosurgery, London, UK; 3 Department of Intensive Care, Royal Free Hospital, London, UK; 4 Department of Acute Medicine, Addenbrooke’s Hospital, Cambridge, UK; 5 Department of Immunology, Oxford University Hospitals, Oxford, UK; 6 Department of Neurology, Royal London Hospital, London, UK; 7 Department of Neurology, Queen’s Hospital, Romford, Essex UK

**Keywords:** Myasthenia, Electromyography, Repetitive nerve stimulation, Antibodies, Congenital

## Abstract

Myasthenia gravis (MG) and congenital myasthenic syndromes (CMS) are a group of disorders with a well characterised autoimmune or genetic and neurophysiological basis. We reviewed the literature from the last 20 years assessing the utility of various neurophysiological, immunological, provocative and genetic tests in MG and CMS. Diagnostic sensitivity of repetitive nerve stimulation test ranges between 14 and 94% and specificity between 73 and 100%; sensitivity of single-fibre EMG (SFEMG) test ranges between 64 and 100% and specificity between 22 and 100%; anti-acetylcholine receptor (AChR) antibody sensitivity ranges from 13 to 97% and specificity ranges from 95 to 100%. Overall, SFEMG has the highest sensitivity while positive anti-AChR antibodies have the highest specificity. Newer testing strategies that have been investigated over the last couple of decades include ocular vestibular-evoked myogenic potentials, otoacoustic emissions and disease-specific circulating miRNAs in serum for autoimmune myasthenia, as well as next-generation sequencing for genetic testing of CMS. While there has been significant progress in developing newer testing strategies for diagnosing MG and CMS over the last couple of decades, more research is needed to assess the utility of these newer tools regarding their sensitivity and specificity.

**Supplementary Information:**

The online version contains supplementary material available at 10.1007/s00415-022-10986-3.

## Introduction

Disorders of the neuromuscular junction comprise a wide range of conditions from myasthenia gravis (MG) to congenital myasthenic syndromes (CMS). Antibodies, genetic mutations, specific drugs or toxins can interfere with the number or functioning of one of the essential proteins that control signalling between the presynaptic nerve ending and the postsynaptic muscle membrane and cause a myasthenic syndrome. In autoimmune MG, antibodies to acetylcholine receptor (AChR) or to proteins involved in receptor clustering, particularly muscle-specific kinase (MuSK) cause direct loss of acetylcholine receptors or interfere with the agrin-induced acetylcholine receptor clustering necessary for efficient neurotransmission [1]. CMS are a genotypically and phenotypically heterogeneous group of disorders, which share an underlying pathophysiology of impaired neuromuscular junction transmission [2]. In clinical settings, autoimmune MG is diagnosed either by antibody testing or by electro-diagnostic testing that shows either decremental compound muscle action potentials in response to repetitive nerve stimulation (RNS) or increased synaptic jitter measured by single-fibre electromyography (SFEMG) [3]. CMS in addition to neurophysiological studies also requires genetic testing for confirmation of its molecular aetiology with most cases being accounted for by mutations in *CHRNE*,* RAPSN*,* COLQ*,* DOK7*,* CHAT* and *GFPT1*.

## Bedside tests

Bedside neuromuscular examination to assess muscle fatigability is the initial step in suspected MG cases followed by laboratory diagnostic testing to confirm the clinical suspicion [4].

The tests that can be performed at the bedside are the rest test, sustained upgaze test, heat test and the ice-pack test. The ice-pack test can be done alone or it can be combined with the heat test or the sustained upgaze test (Simpson test, named after the Scottish neurologist John Alexander Simpson). A rest test (also called sleep test) is based on the characteristic finding in MG that symptoms and signs improve after a period of rest. The utility of this test has been evaluated in ocular MG presenting with ptosis. The median improvement of ptosis with the rest test is about 2 mm and with the ice test 4.5 mm [5].

While there are several variations of performing ice-pack tests or heat tests in those who present with ptosis due to ocular MG, latex party balloons can be filled with ice cold water or with water heated to 45 °C for these tests [6]. Each test is performed for 2 min, with ruler measurements and photographs taken of the palpebral aperture before and immediately after each test [6]. The mean improvements in ptosis with the ice, rest, and heat tests reported are 2.3 (± 1.5) mm, 1.3 (± 1.1) mm, and 0.33 (± 1.4) mm, respectively [6]. In broad terms, the ice-pack test is considered positive if there is an improvement of at least 2 mm of margin reflex distance compared to the level of ptosis before the ice pack is applied [7]. Repeating the ice-pack test can increase its sensitivity further by 35% compared to a single ice-pack test [8].

Combining the ice-pack test, with sustained upgaze for 2 min, is more sensitive than the ice-pack test done alone [7]. Simpson’s test refers to an increase in the degree of ptosis on sustained upgaze, indicating levator muscle fatigability [9]. The sensitivity and specificity for diagnosing MG are 28% and 100% for the ice-pack test done alone, and 73% and 97% when combined with sustained upgaze [7].

## Antibody tests

### Anti-AChR antibodies

The first and still the most common antibody test used is the anti-AChR antibody assay. Traditionally these AChR antibodies have been detected using radio-immuno-precipitation assays (RIPA). Some of the patients (10–15% of generalised MG and 50% of ocular MG cases) classified as ‘seronegative’ using RIPA, have low-affinity antibodies to AChR [10] that cannot be detected in standard solution RIPA. However, about two-thirds of sera from patients previously found to be negative for binding AChR in solution, have been found to be positive for anti-AChR antibody using a cell-based assay method in which antibodies to rapsyn-clustered AChR are detected [10]. Clinically they resemble patients with AChR antibodies detected by standard RIPA [11] apart from the fact that patients with antibodies only to clustered AChR have been found to be younger, have milder disease [12] and generally have a good prognosis [13]. Furthermore, cell-based assays can also be useful in the diagnosis of RIPA-negative MG in children [12]. This method is the primary technique for detecting other antibodies such as anti-LRP4 and it may also be used for anti-MuSK antibodies [14].

Patients with positive anti-AChR antibodies generally do not express a single monoclonal antibody population. The heterogeneous nature of the anti-AChR antibody response has led to the categorisation of AChR antibodies into 3 types: binding, blocking, and modulating antibodies [15]. Both binding and blocking antibodies correlate with disease severity in MG [15]. The most sensitive assay in a study analysing serum samples from 41,180 patients (12% of these had AChR antibodies from any one of the above three categories) was the AChR binding antibody assay, which was positive in 4178 (88%) of the 4740 AChR antibody-positive serum samples [16]. Modulating antibodies were detected in 70% (*n* = 3297) of the samples, and blocking antibodies were least prevalent, detected in only 65% (*n* = 3074) of AChR antibody-positive sera [16]. Combining binding and blocking AChR antibody testing identified 97% of the patient population with detectable AChR antibodies [16].

The sensitivity of AChR antibody in adults is 85–90% in cases with generalised MG and 50–70% in cases of ocular MG. It is only 50% sensitive in cases of childhood-onset MG but nearly 100% in cases of thymoma-associated MG. Specificity of anti-AChR for myasthenia is 95–100%.

### Anti-MuSK antibodies

If anti-AChR-binding antibody is negative or the binding antibody titre is < 0.02 nmol/L, the next step is to test for anti-MuSK antibodies although some clinicians will request both antibody tests at the same time at the initial consultation to save time. Traditionally, anti-MuSK antibodies have also been detected by RIPA using directly^125^I-labeled MuSK. They bind to the extracellular Ig-like domains of soluble or native MuSK. They are predominantly in the IgG4 subclass. The alternatives to RIPA are ELISA (enzyme linked immunosorbent assay), FIPA (fluoro-immuno-precipitation assays) and cell-based assays. The sensitivity of anti-MuSK antibodies is 5–70% in generalised MG cases who are anti-AChR antibody-negative [10, 17–19] and generally they are not detected in patients with purely ocular MG. Specificity of anti-MuSK antibodies is very high (94–100%) as few false-positive results have been reported with anti-MuSK antibodies [20–22].

In about 6 to 10% of MG cases, neither AChR nor anti-MuSK antibodies are present in the sera. These cases are labelled double-seronegative MG. They can be tested for anti-LRP4 (LDL-receptor-related protein 4) and anti-striated muscle antibodies.

### Anti-LRP4 antibodies

LRP4 is a transmembrane protein which has an important role in synaptic development and maintenance. It activates MuSK activity and promotes the clustering of AChRs. Anti-LRP4 antibodies are usually tested using cell-based assays but they can also be detected using RIPA and ELISA.

Anti-LRP4 antibodies have a sensitivity of 1–50% in patients with MG [18, 23]. In one study involving MG patients (*n* = 217), patients with other neurological or psychiatric diseases (*n* = 76), and healthy control subjects (*n* = 45), anti-LRP4 antibodies were detected in 11 MG patients without detectable anti-AChR or anti-MuSK antibodies. No healthy control subjects and only 2 of the 76 control patients with neurological disease had anti-LRP4 antibodies [24]. However, there remains some controversy about the pathogenicity of anti-LRP4 antibodies in MG. Some have argued that anti-LRP4 antibodies play a pathogenic role in the dysfunction of the neuromuscular endplate in patients with MG. In one study (*n* = 13) with nearly half of the patients with MG and anti-LRP4 antibodies, the patients’ sera inhibited agrin-induced aggregation of AChRs in cultured myotubes by more than 50% [18, 24]. Others have argued that the lack of influence of anti-LRP4 antibodies on the different neurophysiological parameters in myasthenic patients raises doubts about the pathogenic role of anti-LRP4 antibodies in MG [25].

Furthermore, anti-LRP4 antibodies are not specific for MG as they have also been found in cases of amyotrophic lateral sclerosis or motor neurone disease [26].

### Anti-striated muscle antibodies

Anti-striated muscle antibodies (also called striational antibodies) react with epitopes on the muscle proteins titin and ryanodine (RyR) receptor. They can be detected by ELISA or RIPA and their diagnostic sensitivity for MG is 20–40% for antibodies to titin and about 15% for antibodies to the ryanodine receptor [27]. Of note, antibodies to RyR correlated with the presence of myositis (*p* = 0.03) in one study of 19 patients with thymoma-associated MG [28]. So, this may be marker for a sub-set of patients who may need monitoring and treatment for both MG and myositis.

### Combinations of antibody tests for different epitopes

To overcome the shortcomings of using only anti-AChR antibody tests, which can miss a substantial proportion of cases, some have argued that testing for acetylcholine receptor, acetylcholinesterase, titin and ryanodine receptor antibodies together can offer a better diagnostic method for MG than each antibody test alone [29] but this is not accepted as a standard in routine clinical practice. In a study designed to test for multiple antibodies in 89 MG patients, AChR, acetylcholinesterase, titin and RyR antibodies were detected in 54%, 20%, 64% and 55% of MG patients, respectively. These levels were higher compared to a matched group of patients with other neurological disease (n = 66) and a group of controls (n = 66) [29]. The combination of the four antibodies assays provided 94% sensitivity and 84% specificity for the diagnosis of MG [29].

## Repetitive nerve stimulation (RNS)

### Decremental conduction in myasthenia gravis

RNS (previously called Jolly test, after German neurologist Friedrich Jolly who first described this test) is more sensitive than anti-AChR antibody tests [30]. RNS is typically performed by stimulating a nerve at 2–5 Hz and recording the compound muscle action potential (CMAP) from the corresponding muscle at rest and after stimulation, to look for any decrement, and after 10–15 s of exercise to look for any facilitation. A 10% reduction in the CMAP amplitude comparing the first with the fourth or fifth CMAP in a train has traditionally been accepted as indicative of myasthenia. The two issues that are relevant to the interpretation of the sensitivity of the RNS are the cut-off for determining a positive test and the choice of the nerve-muscle pair analysed. The other issues are patient discomfort and technical difficulty, and we will discuss this further.

RNS showing ≥ 10% decrement has traditionally been used as the cut-off value for diagnosing MG, but this has never been validated, is subject to a sensitivity versus specificity trade-off, and is not specific to MG [31]. Abraham et al. showed that using a decrement cut-off value of 7% for frontalis and 8% for nasalis increased the sensitivities by 6–11% while preserving specificity (95–96%) [32].

Besides the cut-off value used, the results obtained from RNS studies may partly depend on which nerve and muscle pair is stimulated [33]. While any superficial nerve can be tested in studying decremental conduction in MG, the choice of the nerve–muscle pair may depend on the type of MG. The abnormal response in RNS due to fatigability as expected is more widespread in generalised MG, whereas facial muscles are relatively more affected in ocular MG [34]. Niks et al. reported that RNS of the nasalis muscle was more sensitive in oculobulbar MG (100% sensitivity) when compared to hypothenar muscles (20% sensitivity) [35]. On the other hand, in patients with generalised MG, using hypothenar muscles for RNS studies, had a similar yield compared to the nasalis muscle [35]. In a retrospective study of 122 patients with MG and 182 controls, RNS sensitivities for generalised and ocular MG using the traditional ≥ 10% cut-off value were 46% and 15%, respectively, for frontalis muscle recordings, and 35% and 19%, respectively, for nasalis muscle recordings [32]. To increase the diagnostic sensitivity of RNS in MG, bilateral exploration of at least 3 muscles, a facial muscle, trapezius, and anconeus, has been suggested [36]. In one study of 22 patients with MG, by exploring 12 muscles bilaterally, the global sensitivity of RNS was increased to 82%, while specificity was 100%. The sensitivity in the MG subgroups is highest in generalised MG (89%) followed by oculobulbar MG (86%) and least sensitive in ocular MG (67%) [36].

RNS can cause patient discomfort and can be technically difficult. In one study, the highest mean patient discomfort score was with deltoid followed by nasalis and the technical difficulty was maximal in deltoid needing 36% repetitions followed by serratus anterior (33%) [37, 38]. RNS recording over occipitalis muscle provides a reasonable alternative to nasalis muscle stimulation under conditions, such as muscle atrophy, cosmetic surgery, or botulinum toxin application, in which the nasalis muscle is unavailable for use although sensitivity using the occipitalis muscle is slightly lower than for nasalis (50% versus 69%) [39]. In terms of increasing diagnostic yield, RNS from the nasalis 1 min after muscle activation in MG increases sensitivity by only 0–2% compared to testing a second muscle at rest (9–15%) [40].

Double-step nerve stimulation test (DSST) is an RNS technique that is claimed to accurately discriminate MG patients who have normal conventional RNS from control subjects [41], but this would need to be confirmed in larger case series before it is used in routine clinical practice.

Finally, a couple of other points to consider are the roles of RNS in the emergency settings and in prognostication. RNS can be used to obtain quick results with a high sensitivity before starting emergency treatment when a myasthenic crisis is the first presentation of MG (sensitivity > 90%) compared to antibody tests, which can take a couple of weeks to come back with a result [42]. Besides its role as a diagnostic tool, the utility of RNS in prognosticating patients with MG has been investigated. In a study of 77 patients with MG, decreased RNS compound muscle action potential amplitudes in proximal muscles correlated with MG severity determined using the quantitative MG scale scores [43].

## Electromyography (EMG)

Most neurophysiology laboratories will use single-fibre needle electrodes for performing single-fibre EMG (SFEMG) studies to assess neuromuscular transmission, but disposable single use concentric needle electrodes (CEMG) can also be used for SFEMG studies. The relevant issues for the interpretation of SFEMG results which will be discussed further include the cut-off values of jitter used for determining an abnormal test response, the number of muscle fibre pairs tested, the muscles that can be tested, and voluntary versus stimulated muscle contraction.

### Jitter in myasthenia

The safety factor of neuromuscular transmission can be assessed by measuring the neuromuscular jitter, which reflects the time variability of processing in the motor end-plate [44]. Jitter is usually expressed as the mean consecutive difference (MCD) of the inter-potential interval between 20 pairs of muscle fibres, each pair consisting of two muscle fibres adjacent to each other, sharing the same axon and motor unit. Normal MCD has previously been defined as between 10 and 60 microseconds (μs) [45]. Abnormal jitter has variably been defined as mean jitter exceeding 40 μs or 10% of potential pairs having block or jitter exceeding 54 μs [46]. However, some of these jitter parameters can vary from laboratory to laboratory based on the age of the patient and the muscle used to determine normative data [47]. Jitter is increased in any condition with disturbed end-plate function, such as myasthenic syndromes and ongoing re-innervation [44]. As a result, jitter, just like the electro-decrement in RNS studies, is not specific to MG.

### Single-fibre EMG (SFEMG)

Frontalis, orbicularis oculi and extensor digitorum communis (EDC) are commonly tested muscles in SFEMG, although any superficially accessible muscle can be used including the masseter. SFEMG can be performed with voluntary muscle contraction and in those cases where patient cooperation is problematic, such as those who are unconscious or severely weak, it can be performed with the aid of electrical stimulation. Stimulated SFEMG (SSFEMG) in ocular MG shows similar sensitivity (80%) and specificity (97%) levels [48] as compared to that obtained during voluntary muscle contraction [49].

The diagnostic accuracy of SFEMG in MG has been investigated in several studies. In one large study which recruited 348 patients who had SFEMG, 108 of whom finally received a diagnosis of MG, sensitivity was 78% regardless of MG subtype (73% for ocular MG, and 85% for generalised MG) with a specificity of 91% [49]. In another study using the orbicularis oculi muscle for testing, the sensitivity of SFEMG in diagnosing MG (*n* = 54) was 98% (95% CI 0.94–1.02), while the specificity was 70% (95% CI 0.54–0.86), with a positive predictive value of 79% (95% CI 0.74–0.79) and a negative predictive value of 97% (95% CI 0.94–0.99) [50]. However, even SFEMG can have a significant false-negative rate and one could argue this is operator-dependent. Repeat testing when the clinical suspicion of MG remains high can lead to higher diagnostic rate. In one Italian study, the number of positive tests increased from 79 to 91% in patients with ocular MG when repeat testing was performed in 22 of the 165 cases who had initially tested negative [51].

SFEMG can have prognostic value. An abnormal orbicularis oculi SFEMG in patients with seronegative ocular MG was found to have a high predictive value for response to therapy [52]. In a study of 142 consecutive patients with symptoms of ocular MG and negative AChR antibody, orbicularis oculi SFEMG was abnormal in 31 patients and normal in 111 patients. 29 patients with abnormal SFEMG were treated, and 86% of these had a good response [52].

20 pairs of muscle fibres have been used traditionally in SFEMG data analysis. In one study of 94 patients comparing sensitivity of testing 20 pairs of muscle fibres with fewer recorded pairs, 98% of patients had abnormal SFEMG within 17 pairs in ocular MG, or within 15 pairs in generalised MG, thus shortening the test time and decreasing patient discomfort while preserving test sensitivity [53].

Changes in the parameters of jitter measured with SFEMG are reported to predict clinical change in MG with acceptable accuracy. Additionally, response to immunomodulation correlates with change in jitter values post treatment [54, 55]. Absolute and percentage changes in MCD interval differences are equally accurate in predicting clinical change [54]. However, compared with RNS studies which have a relatively high specificity [56], SFEMG suffers from poor specificity for MG. SFEMG using the orbicularis oculi muscle is very sensitive in patients with ptosis, but in patients with isolated diplopia, SFEMG does not exclude MG [51]. Therefore, SFEMG is not a confirmatory test for the diagnosis of MG, but it has a high negative predictive value in identifying patients without MG [50]. This fact is endorsed by the American Association of Electrodiagnostic Medicine (AAEM) quality assurance committee report where RNS and anti-AChR remain the preferred initial tests for MG [57] (Fig. 2). These tests may be complementary to each other as only around 80% of patients with generalised MG have serum antibodies to AChR by the radio-immuno-precipitation assay (RIPA).

### Concentric needle (electrode) for SFEMG (CEMG)

Several groups have used CEMG to study jitter in disorders of neuromuscular transmission [58]. Jitter analysis of the masseter, EDC and frontalis muscles during voluntary contraction is easy to perform in cooperative adults and has shown similar results for both ocular MG (sensitivity 75%) and generalised MG (sensitivity 94%) [59, 60]. In another study of 21 patients with MG, the sensitivity of CEMG for the diagnosis of MG was 67% and the specificity was 96%. The positive and negative predictive values were 0.93 and 0.76, respectively [61].

The normative data for mean jitter values can vary and this will differ from one EMG laboratory to another. In one study (*n* = 33), the receiver operating characteristic curves cut-off point that provided the highest sensitivity without false-positives was 24.7 μs for mean jitter and 33.1 μs for the eighteenth highest value. Sensitivity was 94% for both parameters [62].

Stimulated jitter analysis (Stim-JA) using a concentric needle electrode without need for voluntary activation may be particularly useful in children [63]. In one study of 13 patients with juvenile MG, the electrophysiological parameters of jitter and blocking correlated significantly with Myasthenia Gravis Foundation of America (MGFA) class, whereas grip strength and spirometry did not correlate with MGFA class [64]. Stim-JA has also been used in adults as well. In one study involving 42 adult patients with MG, it showed a sensitivity of 90% compared to 86% positivity rate for anti-AChR antibodies [65].

## Comparisons of RNS and SFEMG in patients with different antibodies

Comparisons between RNS and jitter analysis, between anti-AChR and anti-MuSK antibody-positive MG have shown that RNS is less sensitive (52%) in MuSK patients compared to AChR antibody-positive MG (93%) patients (*p* < 0.01) [66]. Similar results were shown by Padua et al. in their cohort of 52 seronegative (for AChR antibody) patients, 25 whom had MuSK antibodies [67]. Nemoto et al. found positive jitter in 93% of AChR antibody-positive patients but only in 50% of MuSK antibody-positive patients and the extent of jitter was more in AChR antibody-positive MG patients compared with AChR-negative MG patients (MCD: 76 µs in AChR antibody-positive patients, 36 µs in MuSK antibody-positive patients) [68]. In contrast, Nikolic et al. did not find a significant difference in detecting pathological jitter between the two subtypes of MG patients (90% in anti-MuSK patients compared with 93% in anti-AChR patients, *p* > 0.05) [66]. The extent of jitter though may partly depend on the muscles tested [69]. Kuwabara et al. found abnormal jitter in only one of three MuSK antibody-positive patients in the EDC muscle, but all three had increased jitter in the frontalis muscle [69]. By contrast, all the AChR-positive patients (*n* = 11) showed similarly abnormal jitter in the two muscles [69]. Similar results were obtained in another study by Farrugia et al. where the majority of their MuSK antibody-positive patients (*n* = 13) had normal jitter in EDC despite abnormal jitter in orbicularis oculi muscle [70]. Since MuSK antibody patients are thought to have predominant bulbar, facial and neck muscles weakness compared with AChR antibody-positive MG patients [69], when MuSK antibody-positive MG is suspected, SFEMG should be performed in the most prominently affected muscles for greater sensitivity [69]. Results from RNS studies in MuSK antibody-positive cases are similar to the SFEMG studies, showing higher positive rates (86% sensitivity) when facial muscles are tested compared to AChR-antibody-positive (82% sensitivity) MG cases [71]. This again reflects greater propensity for facial muscle involvement in this MuSK antibody-positive cases and emphasises the importance of including facial muscles in RNS protocols when evaluating these patients [71].

### Sensitivity and specificity of different neurophysiological tests

**Fig. 1:**
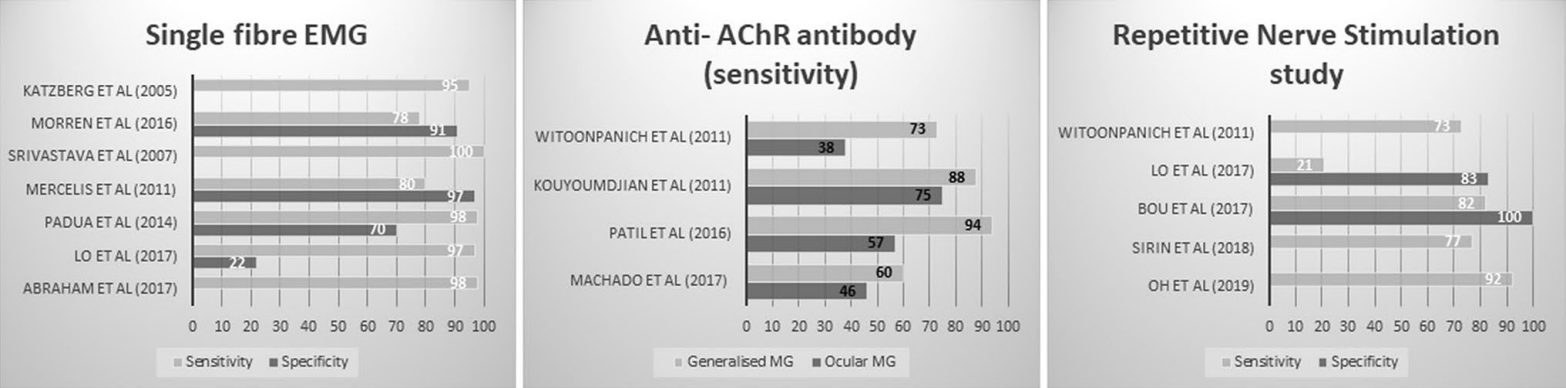
Sensitivities and specificities of various diagnostic tests for myasthenia

While RNS, SFEMG and antibody tests all have their advantages and disadvantages, SFEMG is considered to have the highest sensitivity of all the tests when ordered for diagnostic purposes in both ocular and generalised MG [72], whereas RNS has the highest specificity amongst neurophysiological tests [73] (Fig. 1). The sensitivity of CEMG (93%) is much higher when compared to RNS (77%) in diagnosing MG [74]. Katzberg et al. found 19% of the patients in their study with MG had abnormal RNS, whereas 95% had abnormal SFEMG (*n* = 121) [75].

### Prognostic value of neurophysiological tests

Both RNS and jitter analysis, in addition to their diagnostic utility, may also have a prognostic value as high jitter (> 100 μs) and decrement values (> 10%) are associated with more severe disease, manifested by more frequent symptomatic bulbar and limb muscle weakness and severe disease exacerbations [76]. In contrast, serum concentrations of AChR antibodies do not relate with the clinical severity of MG [77].

## Provocative tests

Provocative tests with quinine or curare are of historical importance only and are not used in clinical practice. The neostigmine test (NT) and edrophonium (tensilon) test are rarely performed in the United Kingdom but are still used in other countries. Although there are several variations of the edrophonium test, the first published protocol was 2 mg initial dose intravenously followed, if no adverse reaction (usually cholinergic side effects) occurred in 45 s, by the administration of an additional 8 mg [78]. One cohort study of the tensilon test reported a sensitivity of 92% for ocular myasthenia and 88% for generalised myasthenia. Specificity was 97% for both types of myasthenia [79]. No statistically significant differences have been found in the edrophonium test results between MuSK antibody-positive and MuSK antibody-negative patients [67]. However, like RNS studies and SFEMG, edrophonium responsiveness is not necessarily diagnostic of MG.

NT is claimed to have a better safety profile when testing patients with MG compared to edrophonium [80], as the latter can cause serious bradyarrhythmias and syncope as well as respiratory failure, although the incidence of these complications is very low [81]. One study involving 47 patients showed the sensitivity of NT to be 83% and specificity to be 97% [82]. An improvement in clinical symptoms in response to NT (0.5 mg neostigmine administered intramuscularly), could be used as supporting evidence for the diagnosis of MG. However, this improvement can be better detected using SFEMG compared to clinical examinations only. A study involving 23 patients revealed SFEMG could better detect subclinical improvement in ocular MG than clinical MG-composite scale. The MCD and potential pairs with increased jitter significantly improved after NT compared to basal conditions [80].

While the NT is claimed to have higher positivity than anti-AChR antibodies and RNS studies [30], neither intra-arterial [83] nor intra-venous NT [84] are commonly used in clinical practice. Edrophonium which is a reversible anti-acetylcholinesterase inhibitor with a short half-life (distribution half-life is 7 to 12 min) had replaced neostigmine (mean half-life of 52 min) for diagnostic testing [85]. However, since 2018, the Food and Drug Administration (FDA) has discontinued edrophonium^®^ as a test for myasthenia, due to its high rate of false-positives and as a result it is no longer commercially marketed in the United States for this purpose [86].

## Ocular vestibular-evoked myogenic potentials (oVEMP)

oVEMP can be used to detect a decrement in the extraocular muscle activity of patients with MG. The protocol involves applying repetitive vibration stimuli to the forehead and recording the activity of the inferior oblique muscle with 2 surface electrodes placed beneath the eyes [87] (Table 1). The decrement over 10 stimulus repetitions at rates 3–20 Hz is recorded.

**Table 1:** Studies showing the sensitivity and specificity of individual tests to diagnose myasthenia

Test	Sensitivity (%)	Specificity (%)	References
Ice-pack test			
All cases of MG	28–96	31–100	[7, 8, 73, 82]
Ocular MG	85	33	[73]
Generalised MG	90	–	[73]
Ice-pack test and SFEMG	79	64	[73]
Ice-pack test and RNS	24	92	[73]
Antibody tests			
Anti-AChR Ab			
All cases of MG	50–97	95–100	[16, 28, 65]
Ocular MG	38–75	–	[30, 60, 72]
Generalised MG	73–94	–	[30, 60, 72]
Clustered AChR Ab	13–66	100	[10–13]
Acetylcholinesterase Ab	19–22	–	[29]
Titin receptor Ab	20–74	–	[27, 29]
Ryanodine receptor Ab	15–74	–	[27–29]
Anti-AChR or Anti-MuSK-Ab	73	–	[74]
Anti-MuSK-Ab	27–70^b^	93–100	[13, 19–22]
Anti-LRP4 Ab	3	–	[13]
All 4 antibodies^a^	94	84	[29]
RNS test			
All cases of MG	15–92	73–100	[31–33, 35, 38, 42, 43, 46, 56, 72–75]
Ocular MG	14–67	91	[30, 31, 36, 60, 65, 72, 73]
Generalised MG	32–94	–	[30, 31, 36, 60, 65, 72, 73]
SFEMG test			
All cases of MG	64–100	22–97	[32, 42, 45, 48–50, 59–61, 65, 73–75, 87]
Ocular MG	73–100	22–100^c^	[32, 49, 52, 60, 62, 72–74]
Generalised MG	85–99	91–100^c^	[32, 49, 60, 62, 72–74]
RNS and SFEMG tests	18	91	[73]
Edrophonium test			
All cases of MG	50–92	97	[67, 79]
Ocular MG	92	97	
Generalised MG	88	97	
Neostigmine test			
All cases of MG	83	97	[82]
Ocular MG	93	–	[30]
Generalised MG	98	–	[30]
oVEMP test	63–89	64–100	[87]

*oVEMP* Ocular vestibular-evoked myogenic potentials, *MG* myasthenia gravis, *SFEMG* single-fibre electromyography, *RNS* repetitive nerve stimulation, *MuSK* muscle-specific kinase, *Ab* antibody, *AChR* acetylcholine receptor, *LRP4* LDL receptor-related protein 4, *RyR* ryanodine receptor

^a^All 4 antibodies = Acetylcholine receptor, acetylcholinesterase, titin and ryanodine receptor antibodies

^b^In those who are seronegative for anti-AChR antibody

^c^Depending on control population

One study compared 27 patients with MG (13 with isolated ocular MG and 14 with generalised MG), with 28 healthy controls. The aim was to identify oVEMP parameters with the highest sensitivity and specificity by evaluating decrement over 10 stimulus repetitions at 3 different repetition rates (3 Hz, 10 Hz, and 20 Hz). Repetitive stimulation at 20 Hz yielded the best differentiation between patients with MG and controls with a sensitivity of 89% and a specificity of 64% when using a unilateral decrement of ≥ 15.2% as cut-off. When using a bilateral decrement of ≥ 20.4% instead, oVEMP allowed differentiation of MG from healthy controls with 100% specificity, but slightly reduced sensitivity of 63%. For both cut-offs, sensitivity was similar in isolated ocular MG and generalised MG [87].

The presence of an oVEMP decrement is reported as a sensitive and specific marker for MG. This test allows direct and non-invasive examination of extraocular muscle activity, with similarly good diagnostic accuracy in ocular and generalised MG [79]. However, the oVEMP has no therapeutic or monitoring value in MG as the results between newly diagnosed patients, patients uncontrolled on treatment and those who are controlled and asymptomatic on treatment, are not statistically different [81]. oVEMP is not used in routine clinical practice for the diagnosis of MG.

## Otoacoustic emissions

Otoacoustic emissions (OAEs) have also been investigated in the diagnosis of MG [88]. Compared with controls, MG patients revealed a significant reduction in the amplitude of transiently evoked OAE (*p* < 0.05) and distortion product OAE at higher frequencies between 2–4 kHz (*p* < 0.05). Further, the OAE alteration significantly correlates with anti-AChR antibody titres in these patients [88].

OAEs are not used in routine clinical practice for the diagnosis of MG.

## Circulating miRNAs

Circulating miRNAs in sera of MG patients have also been investigated as biomarkers in the diagnosis of MG. In a study of 16 MG patients compared with 16 healthy controls, three miRNAs were specifically dysregulated in AChR + MG patient sera samples. Hsa-miR150-5p, which induces T-cell differentiation, as well as hsa-miR21-5p, a regulator of Th1 versus Th2 cell responses, was specifically elevated in sera of MG patients [89]. However, hsa-miR27a-3p, involved in natural killer (NK) cell cytotoxicity, was decreased in MG [89].

Circulating miRNAs are not used in routine clinical practice for the diagnosis of MG.

## Genetic testing for congenital myasthenic syndromes

All the above-mentioned tests can sometimes be technically challenging in newborns suspected to have a CMS. The diagnosis of CMS is typically considered on the clinical basis of fatigable weakness involving ocular, bulbar and limb muscles at birth to early childhood (though adult onset is increasingly recognised), presence of an abnormal neurophysiological study suggestive of a neuromuscular disorder and absence of myasthenic autoantibodies in the sera [2].

While SFEMG may be challenging in newborns due to lack of voluntary muscle contraction on command, SSFEMG can still be performed in those suspected to have a CMS. A newer algorithm, that analyzes the entire SSFEMG waveform, from which cross-correlational coefficients (between 0 and 1.0) are calculated for consecutive pairs of 100 SSFEMG waveforms obtained at each needle position in orbicularis oculi, which are then averaged, has better specificity (87% vs. 53%) but similar sensitivity (88% for both), compared to conventional MCD measurements [90].

Most CMS result from molecular defects in the muscle nicotinic AChR, but they can also be caused by mutations in presynaptic proteins or proteins associated with the synaptic basal lamina, defects in the endplate or defects in protein glycosylation [91]. Sophisticated neurophysiological studies could determine whether the molecular defect lies at the presynaptic terminal or synaptic or post-synaptic terminal; however, apart from the standard tests (RNS showing decrement, SFEMG showing jitter), these are not performed in routine clinical practice.

Furthermore, next-generation sequencing (NGS) panel testing of the genes involved in CMS is now commercially available. In those cases of CMS that remain undiagnosed through to their adult years, because of milder symptoms in early life, refractoriness to acetylcholinesterase inhibitor and immunotherapy should prompt CMS as a differential diagnosis and genetic testing should be considered [92]. A precise genetic diagnosis is important for therapeutic reasons, as treatments that are effective can be different in the various CMS. The genetic basis of the various types of CMS is outlined in Supplementary Table 1.

The search for an aetiology at a molecular level enables further characterisation of subgroups of CMS as a significant number of CMS patients may present in the neonatal period with variable clinical expression [93]. Genetic testing using NGS may be a more convenient and accurate way to establish the molecular diagnosis in these cases. One shortcoming of this approach is that the genetic panel may not include all the 32 genes implicated in CMS and there is also the issue that such testing is not available in all countries.

## Proposed scheme for testing for myasthenia in adults

**Fig. 2:**
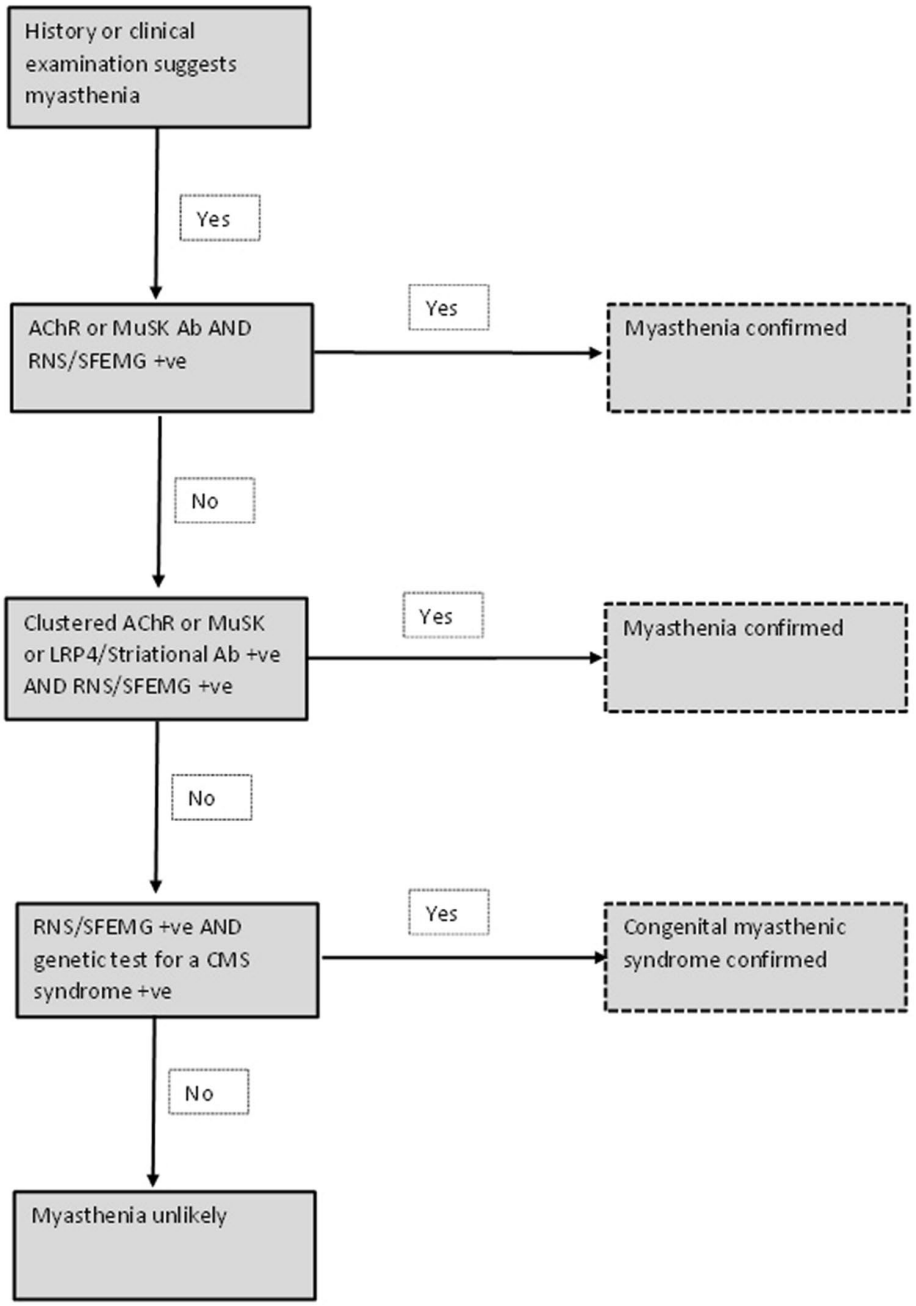
This is a proposed scheme for testing for myasthenia in adults (*RNS* Repetitive nerve stimulation study, *SFEMG* single-fibre electromyography study, *CMS* congenital myasthenic syndrome, *AChR* acetylcholine receptor, *MuSK* muscle-specific kinase, *LRP4* LDL receptor-related protein 4, *Ab* antibody, *+ ve* positive)

Finally, we propose a diagnostic algorithm for testing for myasthenia in adults (Fig. 2) but we accept local protocols may vary. This diagnostic algorithm is based on the fact that combining an antibody test with high specificity and an electro-diagnostic test with high sensitivity is likely to have the highest diagnostic yield. Most clinicians will test anti-AChR antibodies in all suspected cases at initial presentation but the frequency of usage of axonal SFEMG and RNS will depend upon expertise available in the neuromuscular clinic. Where one or both of the neurophysiological tests are positive but the anti-AChR receptor antibody test is negative, most clinicians will test anti-MuSK antibodies because while the neurophysiological tests are very sensitive but they are not specific for myasthenia. If the standard RIPA assays are negative, nowadays clinicians have the ability to test for clustered antibodies against AChR and MuSK using cell-based assays or antibodies to LRP4 or striated muscle. Finally, if the neurophysiological tests are positive for a neuromuscular junction defect but all the antibody tests are negative, one has to consider the possibility that it could be a delayed diagnosis of a milder phenotype of congenital myasthenia that has gone unrecognised up to adult life. Due to the fact that there are more than two dozen genes that can cause congenital myasthenia, with considerable phenotypic overlap, the specific molecular diagnosis is achieved with genetic testing and nowadays for convenience this can be done using panel testing.

## Conclusion

There have been several developments over the last couple of decades in the field of myasthenia from improved immunological testing for anti-AChR antibodies to investigating newer methods, such as oVEMP, OAEs and miRNAs, in research settings for finding better tools for diagnosing MG. NGS genetic panel testing for CMS is now available and the number of genes included in such panels of genes for testing, to elucidate the molecular basis of the several different CMS, will continue to increase in the coming years.

## Supplementary Information

Below is the link to the electronic supplementary material.

Supplementary file1 (PDF 106 KB)
