# Supplementary material for: Bedside and laboratory diagnostic testing in myasthenia
Source: J Neurol. 2022 Feb 10;269(6):3372–84. doi: 10.1007/s00415-022-10986-3 (PMC9119875; doi:10.1007/s00415-022-10986-3)
Supplement: Supplementary file 1 — Supplementary file1 (PDF 106 KB) [file 415_2022_10986_MOESM1_ESM.pdf]

Supplementary Table 1

Genetic testing for congenital myasthenic syndromes will involve one or more of these genes tested individually, sequentially or using panel testing.

Name/Gene ID	Description	Location	Aliases	OMIM
CHRNE	cholinergic receptor nicotinic epsilon subunit	Chromosome 17	ACHRE, CMS1D, CMS1E, CMS2A, CMS4A, CMS4B, CMS4C, FCCMS, SCCMS	100725
CHRNA1	cholinergic receptor nicotinic alpha 1 subunit	Chromosome 2	ACHRA, ACHRD, CHRNA, CMS1A, CMS1B, CMS2A, FCCMS, SCCMS	100690
CHRND	cholinergic receptor nicotinic delta subunit	Chromosome 2	ACHRD, CMS2A, CMS3A, CMS3B, CMS3C, FCCMS, SCCMS	100720
GFPT1	glutamine-fructose-6-phosphate transaminase 1	Chromosome 2	CMS12, CMSTA1, GFA, GFAT,	138292

			GFAT 1, GFAT1, GFAT1m, GFPTL, MSLG, GFPT1	
CHRNB1	cholinergic receptor nicotinic beta 1 subunit	Chromosome 17	ACHRB, CHRNB, CMS1D, CMS2A, CMS2C, SCCMS	100710
MUSK	muscle associated receptor tyrosine kinase	Chromosome 9	CMS9, FADS, FADS1	601296
DOK7	docking protein 7	Chromosome 4	C4orf25, CMS10, CMS1B, FADS3	610285
RAPSN	receptor associated protein of the synapse	Chromosome 11	CMS11, CMS4C, FADS, FADS2, RAPSYN, RNF205	601592
CHAT	choline O-acetyltransferase	Chromosome 10	CHOACTASE, CMS1A, CMS1A2, CMS6	118490
AGRN	agrin	Chromosome 1	AGRIN, CMS8, CMSPPD	103320

LRP4	LDL receptor related protein 4	Chromosome 11	CLSS, CMS17, LRP-4, LRP10, MEGF7, SOST2	604270
COLQ	collagen like tail subunit of asymmetric acetylcholinesterase	Chromosome 3	CMS5, EAD	603033
CHRNG	cholinergic receptor nicotinic gamma subunit	Chromosome 2	ACHRG	100730
AGER	advanced glycosylation end-product specific receptor	Chromosome 6	RAGE, SCARJ1	60021
ALG2	alpha-1,3/1,6-mannosyltransferase	Chromosome 9	CDG1I, CDGIi, CMS14, CMSTA3, NET38, hALPG2	607905
ALG14	UDP-N-acetylglucosaminyltransferase subunit	Chromosome 1	CMS15	612866
COL13A1	collagen type XIII alpha 1 chain	Chromosome 10	CMS19, COLXIIIA1	120350
DPAGT1	dolichyl-phosphate N-acetylglucosaminephosphotransferase 1	Chromosome 11	ALG7, CDG-Ij, CDG1J, CMS13, CMSTA2, D11S366, DGPT, DPAGT, DPAGT2, G1PT, GPT, UAGT, UGAT	191350

GMPPB	GDP-mannose pyrophosphorylase B	Chromosome 3	LGMDR19, MDDGA14, MDDGB14, MDDGC14	615320
LAMA5	laminin subunit alpha 5	Chromosome 20		601033
LAMB2	laminin subunit beta 2	Chromosome 3	LAMS, NPHS5	150325
UNC13A	unc-13 homolog A	Chromosome 19	Munc13-1	609894
MYO9A	myosin IXA	Chromosome 15	CMS24	604875
PLEC	plectin	Chromosome 8	EBS1, EBSMD, EBSND, EBSO, EBSOG, EBSPA, HD1, LGMD2Q, LGMDR17, PCN1, PLEC1b, PLTN, PLEC	601282
PKNOX1	PBX/knotted 1 homeobox 1	Chromosome 21	PREP1, pkonx1c	602100
SCN4A	sodium voltage-gated channel alpha subunit 4	Chromosome 17	CMS16, HOKPP2, HYKPP, HYPP, NAC1A, Na(V)1.4, Nav1.4, SkM1	603967

SLC18A3	solute carrier family 18 member A3	Chromosome 10	CMS21, VACHT	600336
SLC25A1	solute carrier family 25 member 1	Chromosome 22	CMS23, CTP, D2L2AD, SEA, SLC20A3	190315
SLC5A7	solute carrier family 5 member 7	Chromosome 2	CHT, CHT1, CMS20, HMN7A	608761
SNAP25	synaptosome associated protein 25	Chromosome 20	CMS18, RIC-4, RIC4, SEC9, SNAP, SNAP-25, SUP, bA416N4.2, dJ1068F16.2	600322
VAMP1	vesicle associated membrane protein 1	Chromosome 12	CMS25, SPAX1, SYB1, VAMP-1	185880
SYT2	synaptotagmin 2	Chromosome 1	CMS7, MYSPC, SytII	600104

OMIM= online Mendelian inheritance in man database number
